# Experience obtaining legal abortion in Uruguay: knowledge, attitudes, and stigma among abortion clients

**DOI:** 10.1186/s12905-019-0855-6

**Published:** 2019-12-09

**Authors:** Shelly Makleff, Ana Labandera, Fernanda Chiribao, Jennifer Friedman, Roosbelinda Cardenas, Eleuthera Sa, Sarah E. Baum

**Affiliations:** 1 International Planned Parenthood Federation/Western Hemisphere Region, 125 Maiden Lane, 9th Floor, New York, NY 10038 USA; 2 Ibis Reproductive Health, 1736 Franklin St, Suite 600, Oakland, CA 94612 USA; 3 Iniciativas Sanitarias, Hospital Pereira Rossell, Bulevar Artigas 1550, 16600 Montevideo, CP Uruguay; 4 Hampshire College, 893 West Street, Amherst, MA 01002 USA

**Keywords:** Legal abortion, Abortion services, Abortion stigma, Uruguay, Decriminalization, Multiple abortions, Abortions, Latin America, Client experience, Conscientious objection

## Abstract

**Background:**

The abortion law in Uruguay changed in 2012 to allow first trimester abortion on request. Implementation of the law in Uruguay has been lauded, but barriers to care, including abortion stigma, remain. This study aimed to assess women’s experiences seeking abortion services and related attitudes and knowledge following implementation of the law in Uruguay.

**Methods:**

We interviewed 207 eligible women seeking abortion services at a high-volume public hospital in Montevideo in 2014. We generated univariate frequencies to describe women’s experiences in care. We conducted regression analysis to examine variations in experiences of stigma by women’s age and number of abortions.

**Results:**

Most of the women felt that abortion was a right, were satisfied with the services they received, and agreed with the abortion law. However, 70% found the five-day waiting period unnecessary. Women experienced greater self-judgement than worries about being judged by others. Younger women in the sample (ages 18–21) reported being more worried about judgment than women 22 years or older (1.02 vs. 0.71 on the ILAS sub-scale). One quarter of participants reported feeling judged while obtaining services. Women with more than one abortion had nearly three times the odds of reporting feeling judged.

**Conclusions:**

These findings highlight the need to address abortion stigma even after the law is changed. Some considerations from Uruguay that may be relevant to other jurisdictions reforming abortion laws include: the need for strategies to reduce judgmental behavior from staff and clinicians towards women seeking abortions, including training in counseling skills and empathic communication; addressing stigmatizing attitudes about abortion through community outreach or communications campaigns; mitigating the potential stigma that may be perpetuated through policies to prevent “repeat” abortions; ensuring that younger women and those with more than one abortion feel welcome and are not mistreated during care; and assessing the necessity of a waiting period. The rapid implementation of legal, voluntary abortion services in Uruguay can serve in many ways as an exemplar, and these findings may inform the process of abortion law reform in other countries.

## Background

Abortion is legally restricted throughout much of Latin America. However, a wave of legal and policy changes have fully or partially decriminalized abortion over the last twelve years, including in Chile, Colombia, Mexico City and Uruguay [1, 2]. As more countries reform their abortion laws and implement legal services, lessons can be learned from recently decriminalized contexts. These experiences can provide information about how to structure and implement laws in ways conducive to high-quality, accessible, and non-judgmental abortion service provision. In Uruguay, considered one of the most liberal and least religious countries in Latin America [3], the abortion law changed in 2012 to allow abortion on request in the first trimester [4, 5]. Before the legal change, unsafe abortion was the primary cause of maternal mortality in Uruguay. It was responsible for 28% of maternal deaths nationally from 1995 to 1999 and even higher rates of maternal death among socially and economically vulnerable women, who were more likely to access higher-risk clandestine abortion methods [6].

In response to the prevalence and risks of unsafe abortion in Uruguay, the non-governmental organization *Iniciativas Sanitarias* developed a harm-reduction model to provide women with accurate information and counseling on safe methods of pregnancy termination. The model was first implemented in 2001 in *Hospital de la Mujer-Centro Hospitalario Pereira Rossell* (CHPR), a large hospital in Montevideo, and later expanded to other sites in Uruguay [6–8]. This model, in combination with advocacy by feminist groups, helped pave the way for decriminalization and rapid implementation of legal abortion services in 2012 [4, 5].

The 2012 law requires four visits for a voluntary abortion procedure: first, ultrasound, laboratory tests and confirmation of abortion decision; second, a counseling session with a committee of professionals including a mental health professional, a social worker, and a medical doctor; third, a final confirmation of the woman’s decision and the initiation of the (usually medication-based) procedure; and fourth, follow-up, including contraceptive guidance. There is a mandatory five-day reflection period between the second and third visits [5]. The law also supported the right of obstetrician/gynecologists to conscientious objection, specifically to excuse themselves only from providing abortion services in the third visit [9]. The scope of permissible conscientious objection has since been broadened in a 2015 court case [4, 10, 11].

At the time of the 2012 legal change, most countries in Latin America and the Caribbean outlawed abortion entirely or permitted it on narrow grounds. Only a handful of jurisdictions in the region, including Cuba, Guyana, and Mexico City, allowed abortion without restriction as to reason; Uruguay’s law was therefore an advance for the region [12, 13]. Following implementation of the 2012 law, sexual and reproductive health teams (“SRH teams”) were trained by *Iniciativas Sanitarias* at public, government-run facilities to provide legal abortion services across the country, with regulations mandating medication abortion as the standardized method [4]. In 2014, approximately 99% of legal abortion procedures were completed with abortion pills. In the first two years of the law, 15,996 abortion services were provided through the Uruguayan health system [5].

While the implementation of the abortion law in Uruguay has been lauded, barriers to safe abortion care persist [4, 9, 14]. Such barriers include abortion-related stigma. Abortion stigma can be defined as a shared understanding that abortion is morally wrong and/or socially unacceptable [15] and as “a ﻿negative attribute ascribed to women who seek to terminate a pregnancy that marks them, internally or externally, as inferior to ideals of womanhood” [16]. The mandatory waiting period, obligatory counseling, and conscientious objection may perpetuate stigma or impede timely access to care [9, 10, 17]. In addition, the “repeat abortion prevention policy” implemented at the CHPR, which promotes post-abortion contraception [18], could also have unintentionally contributed to abortion stigma towards women who have more than one abortion. Hoggart et al. argue that referring to “repeat” abortion “carries connotations of ‘repeat offender’, suggests a cycle of repeated risky sexual and contraceptive behaviour and of not learning from previous mistakes” [19]. The stigma associated with abortion can contribute to secrecy, social isolation, fears of judgement, and feelings of shame or guilt among women who seek abortion around the globe; it can also result in delays to care or negative health outcomes, and may exacerbate other barriers experienced by women seeking safe abortion services [4, 15, 16, 20, 21].

This study aimed to assess experiences seeking abortion services, perceptions of stigma, attitudes towards abortion, and knowledge of the abortion law among women who sought abortion care following implementation of legal first-trimester abortion in Uruguay.

## Methods

We conducted a cross-sectional descriptive study, collecting qualitative and quantitative data from women and providers. This paper presents quantitative survey data that were collected between February and October 2014 among women who obtained abortion services at the CHPR hospital in Montevideo. Nearly half of Uruguay’s population of approximately 3.2 million lives in Montevideo [22], and the CHPR was selected as the site for data collection because it is the largest women’s hospital in the country—providing the highest number of abortion services out of any facility [23]. We analyzed quantitative and qualitative data separately; the qualitative findings from this study are published elsewhere and are referenced throughout this paper [17].

Women were recruited for the study through convenience sampling before initiating the third of four mandated visits; a hospital staff member trained in study recruitment invited eligible women to participate and provide their contact information if interested. The research team then contacted these women to schedule interviews, which were carried out after the fourth visit. Women were eligible for the study if they were 18 years of age or older, spoke Spanish, and had completed their abortion services at the participating hospital. A trained interviewer administered the questionnaire in-person in a private room at the clinic immediately following the fourth visit, or by telephone at a time convenient for the client. Immediately prior to initiating the survey, participants provided verbal consent and the informed consent form was signed by the interviewer. The survey was completed by the interviewer on an electronic tablet using the secure, online platform SurveyMonkey (SurveyMonkey Inc., Palo Alto, CA, USA).

The survey was developed and applied in Spanish. Survey topics included sociodemographic information and reproductive history, knowledge and opinion on the current abortion laws in Uruguay, participants’ experiences seeking legal services, disclosure to friends and family, perceptions of Uruguayan women’s experience with and reasons for seeking abortion, and perceptions of community attitudes towards abortion. We also adapted the Individual Level Abortion Stigma (ILAS) scale, a ﻿multidimensional measure of individual-level stigma among women who have had an abortion, which was developed, validated and found reliable in the United States [24]. The ILAS comprises sub-scales that are each correlated with the full scale, measuring four domains of stigma: worries about judgement, isolation, self-judgement and community condemnation [24]. Based on consensus among the research team regarding items applicable for the Uruguayan context, we included two full ILAS sub-scales in the survey: “Worries about judgment” (for example, “People would judge me negatively”) and “Self-judgment” (for example, “I felt ashamed about my abortion”). After completing the survey, participants received a small gift bag of toiletries and an information brochure on sexual and reproductive health as compensation for their participation. This form of compensation is standard practice in Uruguay, and was considered by the local study team not to be coercive of participation but to represent a token of appreciation for the study participants’ time and effort. This study was approved by the Allendale Investigational Review (Old Lyme, CT, USA) and the CHPR Research Ethics Committee.

Survey data were exported to Excel for cleaning and imported into Stata 14.2 (Stata, StataCorp, College Station, TX, USA) for quantitative analysis. Qualitative responses to an open-ended question about the source of judgement in services were coded and tabulated in Excel. We ran univariate frequencies for descriptive and demographic variables as well as items related to knowledge, attitudes, and abortion experiences. We hypothesized that both younger women and women with more than one abortion in their lifetime would have higher levels of self-judgement, higher levels of worry about judgement, and be more likely to experience judgement in services. These hypotheses reflect barriers faced by young people globally when seeking sexual and reproductive health services [25] as well as the potential for intensified stigma among younger women seeking abortion [26] and women who have more than one abortion [19]. To examine the hypothesis about age, we selected women 18–21 years of age to approximate the sample of younger women, as this group represents the quartile of women in this study closest to adolescence. We conducted a linear regression to examine whether age group (18–21/22+) or having more than one abortion (yes/no) were associated with scores on the “Worries about judgement” and “Self-judgement” ILAS sub-scales. We utilized logistic regression to examine whether age group or having more than one abortion were associated with self-reported experiences of judgement (yes/no) in women’s recent abortion service.

## Results

### Sample and participant characteristics

**Fig. 1:**
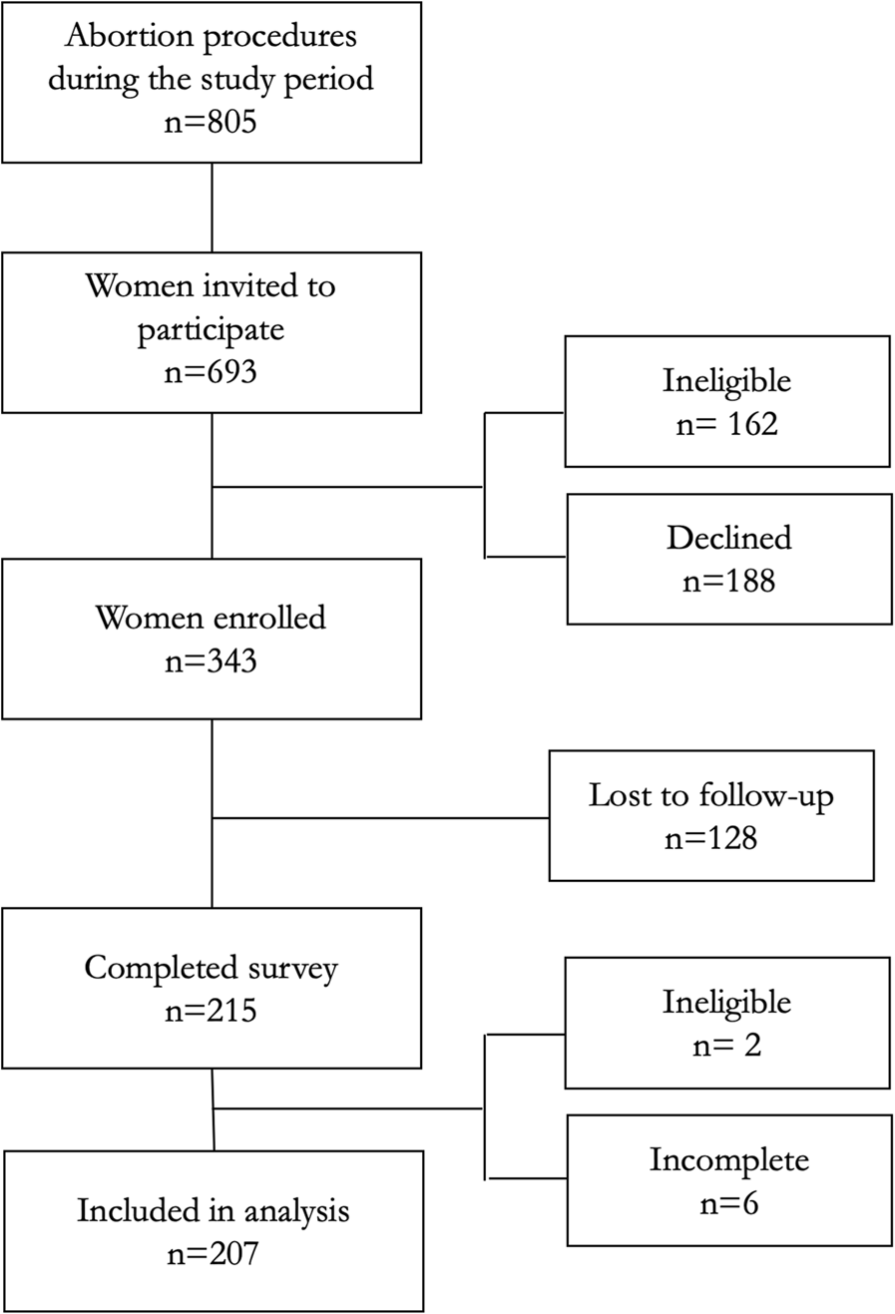
Recruitment of abortion clients in Uruguay

During the study period, 805 women received abortion services at the recruitment hospital. As shown in Fig. 1, a total of 693 women were invited to participate in the study, among whom 162 (23%) were ineligible because they were under 18 years of age and 188 (27%) declined to participate. Therefore, 343 women were enrolled in the study. With 128 women lost to follow-up, we conducted 215 surveys and included 207 women in the analysis after removing those who were ineligible due to age criteria (*n* = 2) or answered less than 15 questions (*n* = 6).

Women in the study had a mean age of 26.7 years (range 18–44), and the majority had not completed high school (73%), were not working (56%), and were partnered or married (58%) at the time of the survey. Seventy-seven percent reported having one or more prior pregnancies and 16% had obtained one or more prior abortions. Slightly more than half were using contraception at the time of the survey (54%) (Table 1). The most common contraceptive methods were condoms and oral contraceptive pills; less than 10% were using long-acting methods such as an IUD or implant (not shown).

**Table 1:** Participant characteristics (*N* = 207)

Characteristics	*n* (%)
Age	
18–21	58 (28.0)
22–25	47 (22.7)
26–30	40 (19.3)
31–35	34 (16.4)
> 35	28 (13.5)
Education	
Less than high school	38 (18.4)
Some high school	114 (55.1)
Completed high school	26 (12.6)
University	27 (13.0)
No response	2 (1.0)
Relationship status	
Single	66 (31.9)
Partner not cohabitating	44 (21.3)
Partner cohabitating	70 (33.8)
Married	7 (3.4)
Separated/Divorced	19 (9.2)
No response	1 (0.5)
Current work	
Informal	42 (20.3)
Formal	48 (23.2)
None	115 (55.6)
No response	2 (1.0)
Prior pregnancies^*^	
0	47 (22.7)
1	42 (20.3)
2	43 (20.8)
3+	74 (35.7)
No response	1 (0.5)
Prior abortion	
Yes	33 (15.9)
No	173 (83.6)
No response	1 (0.5)
Use of contraception at the time of survey	
Yes	112 (54.1)
No, but I would like to	89 (43.0)
No and I would not like to	6 (2.9)

* Includes prior abortions and miscarriages; does not include current pregnancy

## Experience seeking and obtaining abortion services

Women in the study learned about the availability of legal abortion services primarily through television-based news, press releases and other publicity (52%), or from family, friends or acquaintances (23%). Regarding interactions with hospital staff, most of the women reported that they felt they could ask the counselor questions (86%), found it easy to understand the information they received when discussing options for unintended pregnancy (84%), and felt supported by hospital staff in their abortion decision (87%). The majority of women reported being very or somewhat satisfied with the abortion services they received (89%), and that they would recommend these services to friends and family (88%) (Table 2).

**Table 2:** Experience with abortion services (*N* = 207)

Indicators of experience with services	*n* (%)
First source of information about availability of legal abortion	
Institution	16 (7.7)
Friend/Family/Acquaintance	48 (23.2)
Internet	8 (3.9)
Radio	11 (5.3)
Television	108 (52.2)
Other	9 (4.3)
No response	7 (3.4)
Felt could ask questions to the clinic staff	
Yes	178 (86.0)
Somewhat or no	10 (4.8)
No response	19 (9.2)
Ease in understanding information about pregnancy options	
Easy	174 (84.1)
Hard	8 (3.9)
No response	25 (12.1)
Level of support at clinic for abortion decision	
Supported	181 (87.4)
Somewhat or not supported	20 (9.7)
No response	6 (2.9)
Satisfaction with abortion services	
Very satisfied	173 (83.6)
Somewhat or unsatisfied	28 (13.5)
No response	6 (2.9)
Would recommend the abortion services to a friend or family	
Yes	183 (88.4)
No or depends	15 (7.2)
No response	9 (4.3)

### Knowledge and opinion about the abortion law in Uruguay

Information about the abortion law is provided during the first abortion visit. At the time of the survey (after the fourth visit), women were very familiar with the gestational age restrictions specified in the Uruguay law, with 92% identifying the limit at 12 weeks. Most women (69%) also knew that they were required to attend four visits. The conscientious objection component of the law was less clear, with only 23% of women correctly reporting that doctors could object.

Seventy percent of women in the study said they thought it was difficult to access an abortion in Uruguay. The vast majority of women (94%) reported being in general agreement with the current abortion law in Uruguay; however, they had a range of opinions regarding specific components of the law. Regarding gestational age limits, slightly more than half of women (57%) reported supporting a limit at 12 weeks or later, while 40% said women should only be able to have abortions up to 8 weeks of pregnancy. The majority of women (70%) said they found the required five-day waiting period to be unnecessary. Sixty-nine percent of participants reported believing that doctors should have the right to conscientiously object (Table 3).

**Table 3:** Knowledge and opinion about the abortion laws in Uruguay (*N* = 207)

Indicators of knowledge/opinion	*n* (%)
Accurate understanding of components of Uruguay abortion law	
Gestational limit is 12 weeks	191 (92.3)
Four appointments required for abortion process	143 (69.1)
Conscientious objection is permitted	48 (23.2)
Perceived ease of access to abortion in Uruguay	
Easy	39 (18.8)
Hard	149 (72.0)
No response	19 (9.2)
Agreement with the current abortion law in Uruguay	
Yes	195 (94.2)
No	10 (4.8)
No response	2 (1.0)
Acceptable gestational age for a woman to obtain an abortion	
Never	1 (0.5)
8 weeks	82 (39.6)
12 weeks or longer	118 (57.0)
Don’t know	4 (1.9)
No response	2 (1.0
Usefulness of the 5-day waiting period	
Very or somewhat necessary	54 (26.1)
Not necessary	145 (70.0)
No response	8 (3.9)
Agreement with doctor’s right to conscientious objection	
Agree	142 (68.6)
Disagree	50 (24.2)
Don’t know	12 (5.8)
No response	3 (1.4)

### Experiences of stigma throughout abortion process

**Fig. 2:**
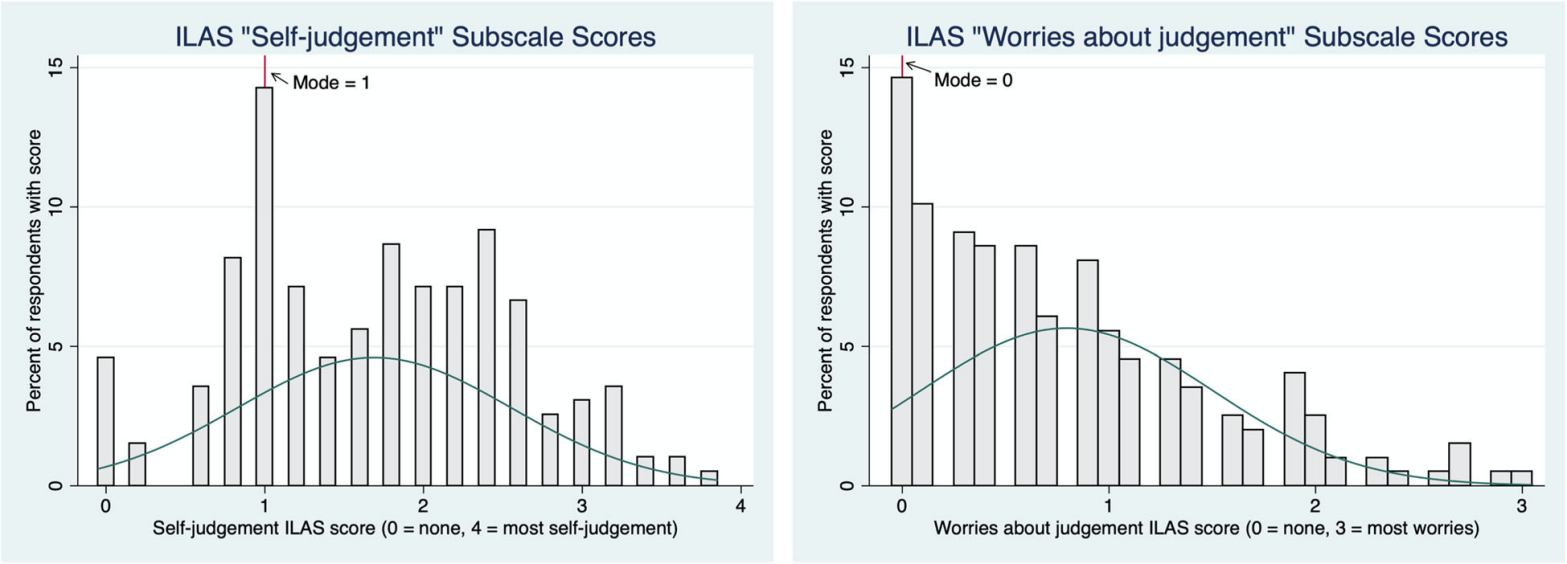
Distribution of scores for two ILAS subscales

Based on scores reported for the two ILAS sub-scales, 95% of women in the study reported some self-judgement and 85% reported some worries about being judged by others (Fig. 2). The distribution of the two ILAS sub-scales suggests that women experienced higher levels of self-judgement compared to worries about judgement. Specifically, the “Worries about judgment” sub-scale (possible score 0–3) had a mode of 0 (reported by 15% of the sample) with a right-skewed distribution, whereas the “Self-judgment” sub-scale (possible range 0–4) had a mode of 1 (reported by 14% of the sample) and was approximately normally distributed. For both, a higher score indicates higher levels of reported stigma (Fig. 2).

We examined whether age was associated with either worries about judgement or self-judgement. Younger women reported more worries about judgment compared to older women. Specifically, the “Worries about judgement” ILAS sub-scale score was 0.31 points lower (CI: = − 0.52, − 0.09, *p* = .006) among women over the age of 21 than among those between 18 and 21 years of age in this sample (0.71 vs 1.02 respectively). There was no significant association between age group and score on the “Self-judgement” ILAS sub-scale (not shown). We also examined whether having more than one abortion was associated with either worries about judgement or self-judgement on the ILAS sub-scales, and found no significant association (not shown).

Twenty-four percent of participants (*n* = 50) reported feeling judged by staff, providers, or other clients while obtaining abortion services. Women described 58 instances where they felt judged: 44% of these instances took place during interactions as part of the general hospital services (for example, while receiving ultrasound, emergency, laboratory, or pharmacy services), 24% were during interactions with members of the SRH team during the first three mandated abortion service visits (by the psychologist, social worker, or gynecologist), and 16% occurred at the reception area (Table 4). Women who had more than one abortion in their lifetime had nearly three times higher odds of feeling judged than women with no prior abortion (OR = 2.93, CI = 1.25–6.85, *p* = 0.013, not shown), after controlling for age, relationship status, and education. When asked about future clinical encounters, 29% of women in this study were worried to some extent (“very worried”, “worried” or “a little worried”) that their doctor would treat them differently if they found out about their abortion.

**Table 4:** Stigma (*N* = 207)

Indicators of stigma	*n* (%)
Felt judged during abortion service	
Yes, by staff/providers/other clients	50 (24.2)
Yes, by self/family	6 (2.9)
No	151 (72.9)
No response	6 (2.9)
Source of judgment experienced during abortion services (*n* = 50)^*^	
Providers/staff in general hospital services	22 (44.0)
Providers/staff in SRH team	12 (24.0)
Providers/staff in admissions/reception	8 (16.0)
Nurse or gynecologist in unspecified team/location	7 (14.0)
Unspecified	6 (12.0)
Other clients	3 (6.0)
Level of worry that doctor would treat differently based on abortion history	
Very worried, worried, or a little worried	60 (29.0)
Not worried	135 (65.2)
No response	12 (5.8)
Agreement with the following statements about abortion	
Abortion is the right of every woman	189 (91.3)
A woman should keep her abortion a secret	41 (19.8)
Women generally have justified reasons for seeking abortion	162 (78.3)
Women get an abortion due to a lack of responsibility	124 (59.9)

* Excludes 163 women who did not report feeling judged by others during service; numbers do not sum to total as some respondents report multiple sources of judgment

### Own attitudes towards abortion

When asked about abortion in general, the vast majority of women (91%) reported that abortion is the right of every woman and only 20% of respondents said that women should keep their abortion a secret. The majority of women in this study (78%) said that most women in society who seek abortions have justified reasons. However, when asked about specific reasons, 60% agreed with the statement that women frequently get an abortion due to a lack of responsibility (Table 4).

## Discussion

This study examined the experiences of women who obtained legal abortion care through the public sector in Uruguay following decriminalization. The vast majority of women felt that abortion should be the right of every woman, yet most believed it was still difficult to obtain. Some women feared judgement from providers before they arrived at care, and others felt negatively towards themselves or other women who seek abortions. Abortion stigma has not been well documented in Latin America. The data presented here contribute to the field by elucidating the internalized, feared, and enacted stigma experienced by women seeking legal abortion services in Uruguay, as well as their beliefs about the abortion law.

While most participants in this study reported receiving non-judgmental abortion care, nearly one quarter of women said they felt judged by a hospital staff member during their recent service. They reported experiencing this judgement while receiving services and at the reception. A qualitative study in 2014 at the same hospital in Uruguay also found that hospital staff can perpetuate stigma and obstruct access to care; this applies in particular to staff who are not on SRH teams, such as sonographers [17]. Research in Colombia similarly found that women seeking legal abortion services may fear and experience mistreatment and stigma [27], and nearly one third of women in a study in Cape Town, South Africa, reported seeking abortion care outside the formal care sector due to worries about stigma and mistreatment from health care providers [28]. These findings indicate the importance of sensitizing staff across administrative and service provision teams to reduce their discriminatory behavior towards women seeking care. This is particularly relevant given the model of public sector service provision in Uruguay, which integrates abortion care with other services in a hospital setting. This model requires that women interact with hospital staff, such as ultrasound technicians or receptionists, who do not work exclusively on abortion care and may be less supportive of the right to abortion. In 2013, *Iniciativas Sanitarias*, through an agreement with the Ministry of Health and the Administration of State Health Services, implemented training and sensitization with hospital staff in the public sector in Uruguay. They changed hospital protocol to limit the interaction of abortion clients with personnel external to abortion provision. Periodic assessments of women’s experiences in care can inform the development of additional strategies to mitigate the risk of enacted stigma towards women who seek abortion.

It is noteworthy that women in this study who had more than one abortion in their lifetimes had three times the odds of feeling judged while obtaining abortion care than those seeking their first abortion. A qualitative study in the same hospital in 2014 found unfavorable attitudes towards women who have more than one abortion among both health professionals and abortion clients [17]. These qualitative findings together with our quantitative results suggest that stigma towards women with more than one abortion may have affected women seeking care at the CHPR at the time of the study. Given concerns that a focus on preventing “repeat abortion” carries with it negative judgement of abortion, it may be that the “repeat abortion prevention policy” implemented at the CHPR exacerbated this particular aspect of abortion-related stigma. This policy, which entails provision of post-abortion contraception, is still being implemented at the hospital. While this is a common public health approach, the framing around preventing “repeat abortion” may contribute to abortion stigma [19], in Uruguay and elsewhere.

Most women in our study expressed perceived stigma in the form of concerns about being judged by others in their community for having an abortion. The data suggest that younger women were more worried about being judged than older women. This is similar to findings in Nigeria that younger women have higher levels of individual-level abortion stigma [26]. Studies in different contexts have also found that women anticipate judgement for seeking abortion, and that fear of judgment may impede access to safe abortion care [17, 20, 29, 30]. Healthcare facilities can play a role in supporting women who experience stigma or anticipate judgement [29]. For example, counseling can help women address feelings of self-judgement and identify coping strategies. In addition, information, education and communication activities could convey the message that everyone is welcomed for non-judgmental care and that young people have the right to equal access to health services by law.

Women in this study also tended to have moderate to high levels of internalized stigma in the form of self-judgment or feeling guilty about having an abortion. Some also expressed negative attitudes towards women who have abortion, by, for instance, saying that they were irresponsible. These findings are consistent with qualitative results from the same hospital in Uruguay indicating that women felt guilt and other negative feelings about their own abortion; they also strongly judged other women who sought abortion, particularly those with more than one abortion [17].

This was the first time, to our knowledge, that the ILAS was adapted for abortion clients in Latin America. While our findings may not be directly comparable due to differences in context, it is noteworthy that the mean score was higher (indicating greater stigma) in Uruguay on both sub-scales compared to findings from the United States in 2011 [24]. The internalized and perceived stigma measured by the ILAS are likely influenced by social norms. In this study, perceived and internalized stigma in care were more commonly reported than enacted stigma. This may reflect that health care providers had already been trained to provide accurate information and counseling on safer methods of pregnancy termination through the harm-reduction model implemented at the CHPR since 2001. As such, hospital staff had already been sensitized on the topic of safe abortion for 11 years before decriminalization, whereas the women in this study may have only encountered public discourse about the topic in the short period since decriminalization. Because it takes time to shift social norms, hospital staff likely had more opportunity for gradual change in their beliefs about abortion, whereas the general population in Uruguay was still early in that process. In their 2014 qualitative study in the CHPR, Cárdenas et al. found that both abortion clients and providers believed that the legal change had favorably influenced Uruguayan perspectives about abortion [17]. As time passes after the legal change, social norms in Uruguay may continue to gradually shift in favor of abortion access, as has been shown in Mexico City [31]; this may eventually reduce women’s experiences of stigma when seeking care. Future studies could explore whether and why women’s experience of abortion-relation stigma have changed over time in Uruguay.

While women in this study tended to agree with the abortion law in general, some disagreed with particular components. Many found the five-day waiting period to be unnecessary; these findings are similar to studies in the United States where women reported little conflict in their decision to seek abortion and highlighted potential negative effects on their emotional well-being as a result of the waiting period. One study in the United States found that waiting periods can increase logistical and financial barriers to care [32]. A substantial group of women in our study (40%) felt that the gestational age limit should be lower than the current limit, which is consistent with findings in other contexts that women who seek abortion care may nonetheless support limiting access to this service for others [33]. The survey did not ask participants the gestational age at the time of their own abortion, which limits analysis of variations in their attitudes by this indicator. However, we postulate that the support among some participants of an earlier gestational age limit in Uruguay relates to their experiences of internalized stigma or judgment towards other women who seek abortion, as described above.

While few participants in this study knew that conscientious objection was legal, the majority of women believed it should be permitted. Conscientious objection and refusal by physicians can have consequences on women’s access to services despite decriminalization, particularly in areas with limited abortion providers such as outside of metropolitan areas. For example, while most hospitals in Uruguay report compliance with the current law, all gynecologists in one province objected after the law changed, essentially denying access to women who could not travel to another province [9]. Majority support for the general concept of conscientious objection even among abortion clients points to the importance of establishing strong referral networks in case of refusals. Additional research is currently being conducted on conscientious objection in Uruguay from the perspective of providers; however, further research is needed on client perceptions about this topic in different contexts.

This study has some limitations. First, the data presented here were gathered in 2014, just after decriminalization, and may not reflect current experiences with or attitudes towards abortion in Uruguay. Second, this study describes women’s beliefs about their abortion immediately after their service but does not address whether and how these may shift as time passes after their abortion. Third, the survey did not gather data on participant religion, religiosity, or gestational age at abortion, which might have helped us better understand the factors associated with different beliefs about abortion. Fourth, only women 18 years of age or older were eligible for this study for ethical reasons, thus, we did not capture the experiences of those younger than 18 years. In addition, over one third of recruited clients were lost to follow-up. While this level of loss to follow-up is within the expected range for clinical or public health studies, women who did not participate in follow-up interviews may systematically differ from those who did, which may bias the findings and conclusions of the paper.

## Conclusions

Even when abortion is legally available and considered by many as a right, as in the case of Uruguay, there remains a need to work towards its social acceptability. This endeavor may take time and effort as it entails working both within health care settings and on community norms. Learning about women’s abortion experiences is a necessary step towards identifying potential areas of intervention that service-delivery organizations can employ to improve quality and ensure the availability of non-judgmental and client-centered services, particularly for the most vulnerable or stigmatized such as younger women and those who have more than one abortion. Future research could inform stigma-reduction strategies by exploring changes over time in women’s experiences of stigma when seeking legal abortion care following decriminalization of the practice. Some considerations from Uruguay in the period after decriminalization that may be relevant to other jurisdictions reforming and implementing abortion laws in the future include: the need for strategies to reduce discriminatory behavior from staff and clinicians towards women seeking abortions, including training in counseling skills and empathic communication; the importance of addressing self-stigma as well as stigmatizing attitudes towards others who seek abortion, for example through community outreach and education activities or communications campaigns; awareness of the potential stigma that may be perpetuated through hospital or government policies aiming to prevent “repeat” abortions; consideration of how to ensure that younger women and those who have more than one abortion feel welcome and are not mistreated during care; and assessment of the necessity of a waiting period. The rapid implementation of legal, voluntary abortion services in Uruguay can serve in many ways as an exemplar, and these findings may inform the process of abortion law reform in other countries as they consider training needs for providers, interventions to support women, and strategies to address potential stigma within legal abortion provision.

## Data Availability

The datasets generated and/or analyzed during the current study are not publicly available due to the confidentiality required by our ethics approval but are available from the corresponding author on reasonable request. The instrument is available on request to the authors as well.

## Abbreviations

CHPR: Hospital de la Mujer-Centro Hospitalario Pereira Rossell
ILAS: Individual Level Abortion Stigma scale
SRH: Sexual and reproductive health

## References

[1] Berer M. Abortion law and policy around the world: in search of decriminalization. Health Hum Rights [Internet] 2017;19(1):13–27. Available from: https://www.ncbi.nlm.nih.gov/pmc/articles/PMC5473035/pdf/hhr-19-013.pdf. PMC5473035 28630538

[2] Official Gazette of Chile. Law number 21,030. 2017.

[3] Kulczycki A (2011). Abortion in Latin America: changes in practice, growing conflict, and recent policy developments. Stud Fam Plan.

[4] Wood S, Abracinskas L, Correa S, Pecheny M (2016). Reform of abortion law in Uruguay: context, process and lessons learned. Reprod Health Matters.

[5] Fiol V, Rieppi L, Aguirre R, Nozar M, Gorgoroso M, Coppola F (2016). The role of medical abortion in the implementation of the law on voluntary termination of pregnancy in Uruguay. Int J Gynecol Obstet.

[6] Labandera A, Gorgoroso M, Briozzo L (2016). Implementation of the risk and harm reduction strategy against unsafe abortion in Uruguay: From a university hospital to the entire country. Int J Gynecol Obstet.

[7] Matía MG, Trumper EC, Fures NO, Orchuela J (2016). A replication of the Uruguayan model in the province of Buenos Aires, Argentina, as a public policy for reducing abortion-related maternal mortality. Int J Gynecol Obstet.

[8] Erdman JN (2015). Access to information on safe abortion: a harm reduction and human rights approach. Harv J Law Gend.

[9] Coppola F, Briozzo L, Nozar F, Fiol V, Greif D (2016). Conscientious objection as a barrier for implementing voluntary termination of pregnancy in Uruguay: gynecologists’ attitudes and behavior. Int J Gynecol Obstet.

[10] Berro PL Legal barriers to access abortion services through a human rights lens: the Uruguayan experience. Reprod Health Matters. 2018;26(52). 10.1080/09688080.2017.1422664 29338662

[11] Tribunal de lo Contencioso Administrativo (High Court of Administrative Affairs). Alonso, Justo y otros con Poder Ejecutivo. Acción De Nulidad. Decision 586. 2015.

[12] Center for Reproductive Rights. The World’s Abortion Laws 2014 [Internet]. 2014 [cited 2018 May 15]. Available from: https://www.reproductiverights.org/sites/crr.civicactions.net/files/documents/AbortionMap2014.PDF.

[13] Center for Reproductive Rights. Abortion Worldwide: 20 Years of Reform [Internet]. 2014 [cited 2018 May 15]. Available from: https://www.reproductiverights.org/sites/crr.civicactions.net/files/documents/20Years_Reform_Report.pdf.

[14] Briozzo Leonel, Gómez Ponce de León Rodolfo, Tomasso Giselle, Faúndes Anibal (2016). Overall and abortion-related maternal mortality rates in Uruguay over the past 25 years and their association with policies and actions aimed at protecting women's rights. International Journal of Gynecology & Obstetrics.

[15] Cockrill K, Herold S, Upadhyay U, Baum S, Blanchard K, Grossman D. Addressing Abortion Stigma Through Service Delivery. 2013.

[16] Kumar A, Hessini L, Mitchell EMH (2009). Conceptualising abortion stigma. Cult Health Sex.

[17] Cárdenas R, Labandera A, Baum SE, Chiribao F, Leus I, Avondet S (2018). “It’s something that marks you”: abortion stigma after decriminalization in Uruguay. Reprod Health.

[18] Nozar F, Greif D, Coppola F, Fiol V, Briozzo L (2016). Legal termination of pregnancy as an opportunity for expanding postabortion contraception: experience at the Pereira Rossell hospital, Montevideo. Uruguay Int J Gynecol Obstet.

[19] Hoggart L, Newton VL, Bury L (2017). “Repeat abortion”, a phrase to be avoided? Qualitative insights into labelling and stigma. J Fam Plan Reprod Heal Care.

[20] Coast E, Murray SF (2016). “These things are dangerous”: Understanding induced abortion trajectories in urban Zambia. Soc Sci Med.

[21] Kumar A (2013). Everything is not abortion stigma. Womens Health Issues.

[22] Instituto Nacional de Estadística, Uruguay. Censos 2011 [Internet]. Montevideo; 2011. Available from: http://www.ine.gub.uy/web/guest/censos-2011.

[23] Schroffel HK. Women’s satisfaction with decriminalized abortion Services at Uruguay’s National Women’s hospital Pereira Rossell [internet]. Emory University; 2015. Available from: https://paa2015.princeton.edu/papers/153217

[24] Cockrill Kate, Upadhyay Ushma D., Turan Janet, Greene Foster Diana (2013). The Stigma of Having an Abortion: Development of a Scale and Characteristics of Women Experiencing Abortion Stigma. Perspectives on Sexual and Reproductive Health.

[25] Mejía ML, Montoya P, Blanco AJ, Mesa ML, Moreno D, Pacheco CI. Barreras para el acceso de adolescentes y jóvenes a servicios de salud [Internet]. 2010. Available from: https://colombia.unfpa.org/sites/default/files/pub-pdf/BarrerasJovenesWeb%281%29.pdf.

[26] Oginni Ayodeji, Ahmadu Sikiratu, Okwesa Nkiruka, Adejo Isaac, Shekarau Hauwa (2018). Correlates of individual-level abortion stigma among women seeking elective abortion in Nigeria. International Journal of Women's Health.

[27] Depiñeres T, Raifman S, Mora M, Villarreal C, Foster DG, Gerdts C (2017). “I felt the world crash down on me”: Women’s experiences being denied legal abortion in Colombia. Reprod Health.

[28] Gerdts C, Raifman S, Daskilewicz K, Momberg M, Roberts S, Harries J (2017). Women’s experiences seeking informal sector abortion services in Cape Town, South Africa: a descriptive study. BMC Womens Health.

[29] Makleff S, Wilkins R, Wachsmann H, Gupta D, Wachira M, Bunde W, et al. Exploring stigma and social norms in women’s abortion experiences and their expectations of care. Sex Reprod Heal Matters. 10.1080/26410397.2019.1661753 PMC7887901 31551027

[30] Shellenberg Kristen M., Moore Ann M., Bankole Akinrinola, Juarez Fatima, Omideyi Adekunbi Kehinde, Palomino Nancy, Sathar Zeba, Singh Susheela, Tsui Amy O. (2011). Social stigma and disclosure about induced abortion: Results from an exploratory study. Global Public Health.

[31] Wilson KS, Garcia SG, Olavarrieta CD, Villalobos-Hernandez A, Rodriguez JV, Smith PS (2011). Public opinion on abortion in Mexico City after the landmark reform. Stud Fam Plan.

[32] Roberts Sarah C.M., Turok David K., Belusa Elise, Combellick Sarah, Upadhyay Ushma D. (2016). Utah's 72-Hour Waiting Period for Abortion: Experiences Among a Clinic-Based Sample of Women. Perspectives on Sexual and Reproductive Health.

[33] Cockrill K, Nack A (2013). “I’m not that type of person”: managing the stigma of having an abortion. Deviant Behav.

